# Supplementary material for: Staphylococcal Resistance Patterns, blaZ and SCCmec Cassette Genes in the Nasopharyngeal Microbiota of Pregnant Women
Source: Int J Mol Sci. 2023 Apr 28;24(9):7980. doi: 10.3390/ijms24097980 (PMC10178740; doi:10.3390/ijms24097980)
Supplement: Supplementary file 1 [file ijms-24-07980-s001.zip › ijms-2284068-supplementary.pdf]

International Journal of *Molecular Sciences*

MDPI

Supplementary Materials:

Table S1. Primer sequences and amplification conditions in PCR reactions for the detection of *blaZ*, *mecA* and *mecC* genes used in this study.

Gene	Primer sequences (5′→ 3′)	Amplicon size (bp)	Reference
blaZ	TGCTGATAAAAGTGGTCAAGCA<br>ACACTCTTGGCGGTTTCACT	165	[12]
mecA	TCCAGATTACAACTTCACCAGG<br>CCACTTCATATCTTGTAACG	162	[27]
mecC	GAAAAAAAGGCTTAGAACGCCTC<br>GAAGATCTTTTCCGTTTTCAGC	138	

Table S2. SCC*mec* cassette genes used in this study based on [28].

Reaction	Gene or gene allele detected	Type/class of *ccr* complex	Amplicon size (bp)
M-PCR 1	*mecA*	II	286
	ccrA1-ccrB	I	695
	ccrA2-ccrB	II	937
	ccrA3-ccrB	III	1791
	ccrA4-ccrB4	VI	1287
	ccrC	V	518
M-PCR 2	*mecA-mecI*	II	1963
	mecA-IS*1272*	I	2827
	mecA-IS*431*	V	804
M-PCR 3	E007 in type I SCC*mec*	I	154
	CQ02 in type IV (IVa) SCC*mec*	IV	458
	M001 in type IV (IVb) SCC*mec*	IV	726
	CR008 in type IV (IVc) SCC*mec*	IV	259
	CD002 in type IV (IVd) SCC*mec*	IV	1242

Abbreviations: M-PCR 1-3 - multiplex-PCR reactions 1-3

Table S3. The frequency of the individual genes detected in this study in relation to the staphylococcal *mecA*-positive species and the number of isolates obtained from a pregnant patient.

Patient no.	Age	Trimester	Antibiotics use history	No. of staphylococcal strains isolated	No. of strain	Species	MALDI-TOF MS identification		Resistance profile	Beta-lactam genes			Type of *ccr* complex	Class of *mec* gene complex	SCC*mec* cassette	Type according to [14]
							score index	±SD		*blaZ*	*mecA*	*mecC*				
C20	26	III	very rare	2	C20-1	*S. epidermidis*	2.042	0.045	PNG,FOX,ERY,C	+	+	-	IV	B	VI	VI
C33	27	III	rare	3	C33-1	*S. aureus*	2.248	0.051	PNG,FOX,CIP,TET,SXT	+	+	-	V	-	ND	-
					C33-2	*S. aureus*	2.023	0.010	PNG,TET,C	+	+	-	IV	-	ND	-
c35	28	I	rare	2	C35-2	*S. aureus*	1.963	0.085	PNG,ERY	+	+	-	-	-	ND	-
					C35-3	*S. aureus*	2.004	0.019	PNG,FOX,ERY	+	+	-	IV	-	ND	-
c36	32	II	rare	2	C36-1	*S. aureus*	2.105	0.020	PNG,ERY	-	+	+	-	-	ND	-
c38	32	III	very rare	2	C38-7	*S. aureus*	2.029	0.030	PNG	+	+	-	-	-	ND	-
c39	33	II	very rare	3	C39-2	*S. aureus*	1.962	0.006	PNG,C	+	+	-	-	-	ND	-
c40	30	III	rare	1	C40-1	*S. aureus*	2.121	0.026	PNG,FOX	+	+	-	V	-	ND	-
c49	29	III	very rare	3	C49-2	*S. aureus*	2.301	0.048	PNG,FOX,CIP	+	+	-	I	A	ND	UT5v
c51	24	III	very rare	1	C51-5	*S. hominis*	2.028	0.67	PNG,FOX,TET,ERY,SXT	+	+	-	-	A	ND	-
c52	31	III	very rare	1	C52-7	*S. hominis*	2.193	0.017	PNG,FOX,ERY	+	+	-	I	A	ND	UT5v
c63	29	II	very rare	2	C63-1	*S. hominis*	2.052	0.023	PNG,FOX,TET,ERY	+	+	-	-	-	ND	-
					C63-2	*S. epidermidis*	2.086	0.045	PNG,FOX	+	+	-	IV+V	-	ND	-

Abbreviations: "+/-" – the presence or absence of gene tested, ND - not defined, PNG - benzylopenicillin (1 U), CIP - ciprofloxacin (5 µg), NOR - norfloxacin (10 µg), TET - tetracycline (30 µg), LIN - linezolid (10 µg), ERY - erythromycin (15 µg), C - chloramphenicol (30 µg), SXT - trimethoprim-sulfamethoxazole (1.25 µg -23.75 µg)
